# Incorporating New Guidelines into School-Based Asthma Management

**DOI:** 10.1007/s11882-026-01282-5

**Published:** 2026-06-13

**Authors:** Jessica Michala Jindra, Angela Duff Hogan

**Affiliations:** 1 Department of Pediatrics, Division of Allergy and Immunology, University of Wisconsin School of Medicine and Public Health-Madison, Madison, WI USA; 2 Department of Pediatrics, Division of Allergy and Immunology, Macon and Joan Brock Virginia Health Sciences at Old Dominion University, Eastern Virginia Medical School, Norfolk, VA USA

**Keywords:** School asthma, Asthma action plans, School nurse, GINA updates

## Abstract

**Purpose of the Review:**

The aim of this review is to identify barriers and provide solutions to the implementation of inhaled corticosteroid-formoterol as rescue therapy within schools.

**Recent Findings:**

GINA guidelines in 2019 and NAEPP in 2020 recommended inhaled corticosteroids combined with formoterol for a rescue inhaler over the traditional short-acting beta-agonist.

**Summary:**

The findings discussed in this article describe the barriers to the implementation of inhaled corticosteroids combined with formoterol for a rescue inhaler in school systems. Traditional asthma action plans, school nursing and parental education, and differences in dosing are some barriers identified. This paper provides solutions for the barriers, including updating and individualizing asthma action plans and providing thorough education to all parties on appropriate dosing for ICS-formoterol inhalers.

## Introduction

Asthma is common among school-aged children. They need rescue inhalers readily available for symptoms like cough, wheezing, shortness of breath, or chest tightness. Because children spend much of their time at school, schools must have access to students’ inhalers and ensure their proper use. School personnel, including nurses and teachers, help children monitor symptoms and use inhalers. For decades, short-acting beta-agonists (SABAs) were the standard rescue medication for all ages, with schools following action plans to guide SABA use. However, studies show that a combination of an inhaled corticosteroid (ICS) and formoterol is more effective than SABA alone [1, 2]. Since 2019, the Global Initiative for Asthma (GINA) has recommended ICS-formoterol as rescue therapy for 5-11-year-olds at steps 3 and 4 and for those 12 + at all steps. The 2020 National Asthma Education and Prevention Program (NAEPP) made similar recommendations [3]. These updates have changed traditional action plans, requiring schools to adapt. Schools must adopt new guidelines to provide optimal care.

## Overview of Asthma Guidelines Updates

One in 16 US children has asthma [4]. For over 50 years, short-acting beta-agonists (SABAs) have been the first-line therapy for asthma symptom relief. However, they do not address the underlying type 2 airway inflammation. SABA overuse can downregulate beta-2 (B2) receptors and reduce the bronchodilator response, leading to tolerance [5, 6]. Overuse is also linked to more exacerbations, higher oral corticosteroid (OCS) use, and increased mortality [7, 8]. Corticosteroids are used for exacerbations and as maintenance therapy—specifically, inhaled corticosteroids (ICS). They suppress inflammation, reduce hyperresponsiveness, and lower long-term risks. In 2019, the Global Initiative for Asthma (GINA) recommended ICS–formoterol as the preferred reliever for all steps in children 12+, and for steps 3 and 4 in children aged 6–11 [9]. Used only as a reliever, this approach is Anti-Inflammatory Reliever (AIR); as both a reliever and maintenance, it is Single Maintenance and Reliever Therapy (SMART) or Maintenance and Reliever Therapy (MART).

Using ICS-formoterol as a reliever provides anti-inflammatory dosing with every use. This approach markedly reduces severe exacerbations, even in mild asthma [12]. Multiple studies showed ICS-formoterol was superior to SABA alone, ICS and SABA reliever, and even other ICS-LABAs plus SABA reliever [10, 11]. GINA describes this as the preferred Track 1 regimen [11]. GINA 2025 states, “the safest and most effective approach to asthma treatment in adolescents and adults uses the combination of inhaled corticosteroids and formoterol across all asthma severity levels” [11]. In October 2025, data from the CARE (Children’s Anti-inflammatory Reliever) study showed ICS-formoterol is safe and effective in children as young as 5 years old at steps 1 and 2 of the GINA asthma management Table [12].

In the US, the National Asthma Education and Prevention Program (NAEPP), which published a limited update in 2020, did not eliminate SABA-only (short-acting beta-agonist) use but introduced SMART (single maintenance and reliever therapy) as an option for those inadequately controlled on ICS (inhaled corticosteroids) alone, starting at age 5, for moderate to severe asthmatics [3]. The NAEPP guidelines have not been updated in the last 6 years, and at best, they are now less sweeping and more conservative. With these changes in asthma management, it is important that therapy is consistent across home, school, and extracurricular settings for each child.

## Building Effective School-Based Asthma Care: Required Components and Key Obstacles to Address

**Fig. 1:**
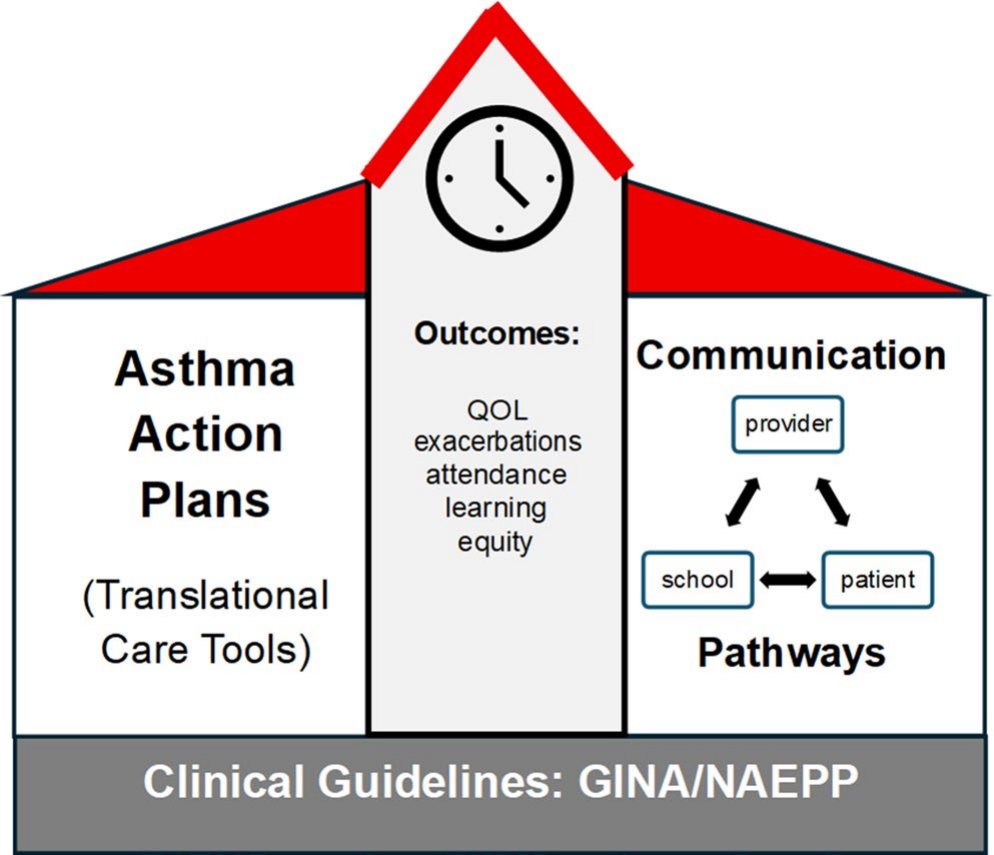
Framework for implementing updated asthma guidelines into school based care

Typical components of school asthma care include asthma action plans (AAPs), medication administration policies, and emergency response measures. GINA 2025 references AAPs 103 times and stresses that every patient should have an AAP appropriate for their health literacy [11]. Written AAPs can be printed, digital, or pictorial, but are essential for providing translational care to help parents and schools appropriately treat children with asthma using the best evidence in asthma care (Fig. 1). Asthma action plans help improve communication between providers, families, and patients. They lead to improved outcomes, better quality of life, fewer exacerbations, improved attendance and learning, and more equitable care [11].

Depending on when a provider sees a patient, AAPs may become outdated midyear. This makes it hard for the nurse to track expiration dates for both medications and action plans. One solution is to date AAPs like sports physicals, using the student’s current academic year (e.g., 2026–2027). Dating the form for both semesters also eases families’ burden.

School nurses and approved personnel have provided asthma care for many years. However, new guidelines add a key component to school asthma policies: effective medication administration (Table 1). Often, medication administration staff are unaware that the 2-in-1 inhaler (ICS-formoterol) can serve as both an asthma reliever and a maintenance medication if needed. There is also confusion about how many pre-exercise or rescue inhalations are needed. ICS-formoterol is dosed as one puff for pretreatment or rescue. SABA, by contrast, is dosed from 2 to 6 puffs depending on the situation and provider [11]. Additionally, SABA is usually dosed every 4–6 h as needed. ICS-formoterol is given as needed, not on an assigned interval. The onset of action for both ICS-formoterol and albuterol is comparable: effects begin within minutes of inhalation, with bronchodilation in 5–15 min.

**Table 1:** Barriers to implementing updated asthma guidelines in schools and potential solutions

Barrier	Impact on Guideline Adherence	Practical Solutions
Confusion about SMART therapy	→ inconsistent rescue treatment	• Simplified Asthma Action Plans • Back-to-School educational resources for schools, useful websites, handouts • Consistent Asthma Action Plans with GINA guidelines
Medication administration due to limited nursing coverage	→ delayed medication administration	• Train all ancillary personnel • Standing orders • Self-carry orders • Stock undesignated inhalers
Legal and policy frameworks	Self-carry laws, stock albuterol Variability across states and school systems	• Know your state’s laws and advocate for policy changes if needed
Equity gaps	Cost and access to care	• Advocate for lower costs of ICS-LABA inhalers • Education of primary care physicians on importance of identifying asthma symptoms and refer when necessary
Outdated AAP/expired medications /lack of spacers	Inadequate rescue treatment	• Update existing AAP to include ICS-LABA therapy • Evaluate for expired medications and provide spacers • Date AAP for academic school year

The maximum recommended daily dosage of albuterol is up to 12 inhalations. Using more than 8–12 puffs per day signals poor asthma control and the need for medical evaluation [11]. IICS-formoterol use is guided by age-specific thresholds for total daily inhalations rather than strict maximum limits. In children aged 6-11 years, use of more than 8 inhalations in 24 hours, and in those aged ≥ 12 years, use of more than 12 inhalations in 24 hours (both maintenance and as-needed doses) means the patient should contact medical providers. Frequent use of ICS–formoterol does not cause tolerance or loss of effectiveness, which may happen with repeated unopposed albuterol use. Some schools and families do not want to use ICS-formoterol as a rescue therapy because it is not FDA-approved for this use, despite support from both asthma guidelines [11, 13].

Many U.S. action plans, including those from the American Academy of Pediatrics (AAP) and the American Academy of Allergy, Asthma & Immunology (AAAAI), still rely on scheduled rescue dosing, such as “every 4 hours.” This differs from the GINA-recommended approach of as-needed reliever use [14, 15]. The American Lung Association action plan allows for separate dosing of ICS-formoterol and SABAs [16]. Although GINA 2025 provides clear criteria for what constitutes a high-quality asthma action plan, it does not offer a standardized template and instead references Australian action plans, such as the nationally endorsed SMART plan. This plan leaves the rescue dosing section customizable, letting clinicians specify individualized as-needed ICS–formoterol instructions [17]. Some U.S. states have begun modernizing their templates. Virginia’s state action plan, for example, includes both ICS–formoterol as reliever therapy and traditional SABA-based tracks [18]. Some large school districts across the country have implemented simplified action plans for schools that list only the rescue inhaler. These plans treat albuterol and ICS–formoterol as interchangeable, commonly recommending two pretreatment puffs before exercise. Rescue puffs are then allowed without distinguishing between the medications; if symptoms persist, staff are told to give four more puffs of the same rescue inhaler. Some states make an undesignated SABA inhaler available to any student who needs bronchodilation. Albuterol in acute settings can help, but it reinforces albuterol use among asthmatics. This may hinder the transition to the single-inhaler, anti-inflammatory reliever strategy promoted by GINA. These differences between national guidance, state policies, and school practices create a key gap. This gap must be addressed to bring improved evidence-based asthma management into U.S. schools.

School nurses and staff are responsible for implementing updated asthma guidelines. Staffing and resources, though, differ greatly among districts. For non-medical staff, simplified, symptom-based instructions are essential. Ongoing education is important not only for school nurses but also for teachers, coaches, administrators, students, and caregivers. This helps ensure safe and consistent asthma management. Pediatricians, subspecialists, families, and schools must communicate effectively. This ensures properly labeled medications, spacers, and current action plans reach the school. Families also face medication expiration issues—albuterol and mometasone-formoterol usually expire 12 months after pharmacy labeling. Budesonide-formoterol is labeled to expire 3 months after opening the foil pouch. Guidance relating to this labeled expiration date will need to be addressed nationwide. Clear processes keep current supplies in schools. These points stress the need for streamlined support and coordinated systems when using new asthma guidelines in schools.

The legal aspects of medication administration at school are also important. Since 2012, most states have passed laws requiring public schools to stock undesignated epinephrine for anaphylaxis. This is not true for albuterol. State laws regarding the stocking and administration of albuterol vary widely, according to the Asthma and Allergy Foundation of America (AAFA). Currently, twenty-five states allow schools to stock emergency asthma medications [19]. These laws differ by state regarding training mandates, liability protections, documentation requirements, and personnel administration. The states that allow usage of stock emergency asthma medicines are Arizona, Arkansas, California, District of Columbia, Florida, Georgia, Illinois, Indiana, Iowa, Kansas, Kentucky, Louisiana, Maryland, Massachusetts, Missouri, Nebraska, New Hampshire, New Mexico, New York, Ohio, Oklahoma, South Carolina, Texas, Utah, Virginia, and Wisconsin. The School-Based Allergies and Asthma Management Program Act (HR 2468), signed in January 2021, encourages states to improve asthma care in schools [19]. It prioritizes federal grants for schools that adopt best management practices. While it does not require stock albuterol, it asks for comprehensive school asthma programs. These programs should: identify affected students, use individualized action plans, ensure staff are trained to give medication, educate school personnel, reduce triggers, and support families. See the wording for each state law at the Asthma and Allergy Foundation of America website [19].

## Ensuring Equitable Asthma Care in Schools: Disparities, Vulnerable Groups, and System Challenges

Implementation of ICS-formoterol therapy comes with several obstacles to ensure equitable care. There are disparities that affect asthmatics across the population. Asthma is more prevalent among black (11.6%) and American Indian/Alaskan (9.2%) native children compared to white, non-Hispanic (5.5%) populations [20]. Although these populations have similar rates of exacerbations (white- 42.7%, black- 39.3%, American Indian/Alaskan- 47.9%), the rates of mortality of black children under 18 years of age are significantly higher than those of their white counterparts, with rates of 7.7% and 1%, respectively [20]. Morbidity and mortality rates are multifactorial, including inadequate diagnosis and inadequate treatment. Families with lower socioeconomic status have been shown to receive fewer referrals to asthma specialty care [20]. These families have higher rates of depression and anxiety [21]. The impact of switching from ICS and SABA to the ICS-formoterol combination can be financially costly. In forty-five states, ICS–formoterol is fully covered across all Medicaid plans [22]. At least 33 states, however, impose restrictive quantity limits (< 3 inhalers/month), meaning only a minority currently allow the 2–3 inhalers per month needed for SMART/MART, though specific counts of “2 inhalers/month” states are not published [16]. Michigan and Virginia are confirmed examples of state Medicaid programs that explicitly cover two ICS–formoterol inhalers per month [23, 24].

Education about SMART/MART use is limited. Many patients do not have routine established care but rather have their asthma treated from one flare to the next. Even when primary care has been established, subspecialty care remains inaccessible. Even among subspecialists, implementation of the GINA guidelines is not universal [25].

A recent national survey of asthma clinicians shows that even specialists have not fully embraced GINA recommendations or SMART therapy. Specialists are more familiar with both NHLBI and GINA guidelines, but cost remains a major barrier to adopting ICS–formoterol reliever therapy. Both specialists and generalists most commonly prescribe albuterol alone. The survey also finds wide variability in definitions of mild asthma, how guidelines are used, and treatment patterns. This variability in clinician practices remains a major barrier to implementing guideline-based care, including in schools.

## Conclusion

ICS-formoterol as a rescue therapy has demonstrated clear benefits in reducing exacerbations and morbidity. For over a decade, its implementation has been slowed by practical and systemic hurdles. ICS-formoterol dosing regimen does not align neatly with traditional asthma action plans. Providers are encouraged to create individualized asthma action plans. Teachers, nurses, and parents are more familiar with the SABA-only rescue approaches, which can lead to incorrect use and dosing. In-depth education for all stakeholders is key to improving asthma care.

Cost and insurance coverage of ICS-formoterol prescriptions are also barriers to implementation. Meaningful policy changes have to be made to improve insurance coverage, allow multiple devices at a time and reduce disparities that disproportionately affect families of lower socioeconomic status. A stronger push toward broader Medicaid coverage of these medications is critical. While steps toward system improvement are underway, our findings show persistent deficits that need immediate attention. Continued commitment, advocacy, and system-level changes are necessary if we are to fully realize the promise of ICS–formoterol rescue therapy and deliver equitable and the best evidence-based asthma care for all patients.

## Key References

- Global Initiative for Asthma. GINA 2025/2026 Strategy Reports [Internet]. 2025/2026 [cited 2026 May 25]. Available from: www.ginasthma.org.
  - ⚬ The GINA 2025/2026 Strategy Reports are the most current, globally recognized sources for evidence-based asthma recommendations. School-based asthma programs should follow these standards of care. These reports guide updates to clinical protocols, medication use, and symptom-response strategies in schools. GINA outlines the latest consensus and best practices in pediatric asthma care. Schools should review their asthma management policies to ensure they align with GINA 2025/2026 recommendations.
- Asthma and Allergy Foundation of America. Asthma Quick-Relief Medicines in Schools [Internet], 2024 [cited 2026 March 30]. Available from: https://aafa.org/advocacy/key-issues/access-to-medications/albuterol-in-schools/.
  - ⚬ This reference outlines how state-level laws shape what schools are currently legally permitted to do in their schools to help children with asthma. It also highlights where policies differ across states, which helps promote practical advocacy efforts to improve school asthma management. This site helps show how updated guidelines can be implemented within real-world legal frameworks. Based on these findings, schools and policymakers can use this website to review their current asthma management policies and align them with best practices reflected in state laws.

## Data Availability

No datasets were generated or analysed during the current study.
